# Abilities of Canine Shelter Behavioral Evaluations and Owner Surrender Profiles to Predict Resource Guarding in Adoptive Homes

**DOI:** 10.3390/ani10091702

**Published:** 2020-09-20

**Authors:** Betty McGuire, Destiny Orantes, Stephanie Xue, Stephen Parry

**Affiliations:** 1 Department of Ecology and Evolutionary Biology, Cornell University, Ithaca, NY 14853, USA; dmo64@cornell.edu; 2 Department of Animal Science, Cornell University, Ithaca, NY 14853, USA; sx228@cornell.edu; 3 Cornell Statistical Consulting Unit, Cornell University, Ithaca, NY 14853, USA; sp2332@cornell.edu

**Keywords:** dog, food aggression, food guarding, resource guarding, animal shelter, behavioral evaluation, adoption, owner surrender

## Abstract

**Simple Summary:**

Some domestic dogs guard resources and display behaviors such as growling, snarling, or biting when approached. Most animal shelters test for food-related aggression and some consider dogs assessed as food aggressive to be unadoptable and candidates for euthanasia. We surveyed adopters of 139 dogs assessed as either resource guarding (*n* = 20) or non-resource guarding (*n* = 119) at a New York (NY) shelter to determine whether shelter identification as food aggressive was associated with guarding in adoptive homes. We also examined whether description of resource guarding in owner reports completed when surrendering a dog to the shelter predicted guarding in adoptive homes. Statistically, shelter assessment as resource guarding and owner-supplied information indicating resource guarding were each associated with guarding in adoptive homes. However, more than half of dogs either assessed by shelter staff or described by surrendering owners as resource guarding did not guard in adoptive homes. Our data indicate that information from surrendering owners, while potentially helpful, is not always predictive of a dog’s behavior in an adoptive home, and most importantly, that shelters should not consider all dogs assessed as resource guarding to be unadoptable because many of these dogs do not display guarding behavior post adoption.

**Abstract:**

Some shelters in the United States consider dogs identified as food aggressive during behavioral evaluations to be unadoptable. We surveyed adopters of dogs from a New York shelter to examine predictive abilities of shelter behavioral evaluations and owner surrender profiles. Twenty of 139 dogs (14.4%) were assessed as resource guarding in the shelter. We found statistically significant associations between shelter assessment as resource guarding and guarding reported in the adoptive home for three situations: taking away toys, bones or other valued objects; taking away food; and retrieving items or food taken by the dog. Similarly, owner descriptions of resource guarding on surrender profiles significantly predicted guarding in adoptive homes. However, positive predictive values for all analyses were low, and more than half of dogs assessed as resource guarding either in the shelter or by surrendering owners did not show guarding post adoption. All three sources of information regarding resource guarding status (surrender profile, shelter behavioral evaluation, and adopter report) were available for 44 dogs; measures of agreement were in the fair range. Thus, reports of resource guarding by surrendering owners and detection of guarding during shelter behavioral evaluations should be interpreted with caution because neither source of information consistently signaled guarding would occur in adoptive homes.

## 1. Introduction

Resource guarding aggression represents a suite of behaviors, such as growling, freezing, snapping, and biting, shown by some domestic dogs that are possessive of food, toys, or sleeping sites [1]. According to one survey of 77 animal shelters in the United States, most shelters test for food guarding during behavioral evaluations and about half consider dogs identified as food aggressive to be unadoptable and therefore candidates for euthanasia [2]. In contrast, successful re-homing of most shelter dogs assessed as food aggressive has been reported, although more than one effort at adoption was sometimes needed because dogs that displayed severe guarding during assessments were returned more frequently by adopters [3]. Even so, shelters that make food aggressive dogs available for adoption often restrict who can adopt them, which can result in longer shelter stays [4,5]. Because resource guarding can affect both public safety and dog welfare, it is important to determine whether dogs assessed as food aggressive during shelter behavioral evaluations display food aggression in adoptive homes. This topic is especially relevant given recent critiques regarding the usefulness and predictive abilities of shelter behavioral evaluations with regard to tests for resource guarding and other behaviors [4,6,7,8].

Four studies, two examining several types of behavior and two focused exclusively on resource guarding, have investigated whether tests conducted under shelter conditions successfully predict behavior in adoptive homes. Van der Borg et al. [9] developed and administered 21 tests to 81 dogs in five different shelters and surveyed adopters after dogs had spent 1–2 months in the home. The authors compared shelter test results with 72 reports from adopters. For aggression displayed over food or a bone, shelter tests were consistent with adopter reports about 43% of the time. Clay et al. [10] examined whether results from 11 tests run at one shelter predicted post-adoption behavior of 120 dogs. Although results from some shelter tests, such as those assessing friendliness, fearfulness, and anxiousness, reliably predicted behavior in the home one month after adoption, results from tests for resource guarding did not (percentage of dogs assessed as food aggressive in the shelter and reported to be food aggressive in the adoptive home was not provided). Mohan-Gibbons et al. [2] identified 96 dogs assessed as food aggressive at one shelter, placed them on a food program (free-feeding and foraging enrichment), and contacted their adopters three times in the months following adoption. Food guarding was rarely reported in the first three weeks in the home: for example, of the 60 adopters who responded at least once, six reported at least one guarding incident. No guarding was reported by these six adopters at 3 months post adoption, although one new incident of guarding was reported by another adopter at this time. Marder et al. [5] followed 97 shelter dogs and compared results from behavioral evaluations at the shelter to adopter reports at least 3 months after adoption. Unlike the dogs followed by Mohan-Gibbons et al. [2], this sample included dogs assessed as food aggressive and dogs assessed as not food aggressive in the shelter, and dogs were not placed on a specific food program. Of the 20 dogs assessed as food aggressive in the shelter, 11 (55%) showed food aggression in the adoptive home. Of the 77 dogs assessed as not food aggressive in the shelter, 17 (22%) showed food aggression in the adoptive home. Thus, in the three studies reporting percentages for resource guarding, the percent of dogs assessed as food aggressive in the shelter that showed food aggression in the adoptive home ranged from about 10% to 55%.

Given the wide range in percentages reported from the three previous studies comparing shelter evaluation results with adopter reports in regard to resource guarding [2,5,9], we revisited this question at one animal shelter in New York. Our study design was most similar to that employed by Marder et al. [5] in that we contacted adopters of dogs assessed as food aggressive in the shelter as well as adopters of dogs assessed as non-food aggressive to determine whether guarding behavior was exhibited in the adoptive home, and dogs in our study were not maintained on a food program. Our study differs from that by Marder et al. [5] in that we contacted adopters 1–3 months post adoption whereas they contacted adopters at least 3 months post adoption and many were contacted one or more years after adoption. Additionally, whereas the adopter survey conducted by Marder et al. [5] focused on food and food-related items (rawhides and bones), we also asked adopters about the guarding of sleeping sites to determine whether dogs that displayed food-related aggression at the shelter would guard non-food items in the home. Finally, unlike the studies by Mohan-Gibbons et al. [2], Marder et al. [5], Van der Borg et al. [9], and Clay et al. [10], we also investigated whether information supplied by surrendering owners predicted behavior in the adoptive home. Specifically, we examined whether owner answers to questions concerning reasons for surrender, resource guarding, and aggression predicted guarding behavior in the adoptive home. Thus, for a subset of dogs, we were able to compare owner surrender profiles, shelter behavioral evaluations, and adopter reports for consistency in assessment of resource guarding.

Based on the evidence presented by Marder et al. [5], we predicted that about half of dogs that showed food-related guarding during shelter behavioral evaluations would show food-related guarding in their adoptive homes, and that about a quarter of dogs assessed as non-food guarding in the shelter would show food-related guarding in their adoptive homes. Given that resource guarding appears sensitive to context, setting, and type of resource [1,2,5], we did not expect an assessment of food-related aggression in the shelter to be associated with the guarding of sleeping sites in the adoptive home. Owners may under-report problematic behaviors at the time of surrendering their dogs to shelters [11], so the predictive ability of owner reports might be lower than that of shelter behavioral evaluations. Alternatively, because shelter evaluations are conducted under conditions that are unfamiliar and often challenging for dogs [6,7], owner reports might better predict behavior in adoptive homes, even though no two homes are identical. We viewed these outcomes as equally likely. Throughout this paper, we use the term “owner” to refer to a person surrendering a dog to the shelter and “adopter” to refer to a person taking a dog home from the shelter.

## 2. Materials and Methods

### 2.1. Study Shelter

We analyzed canine surrender profiles and shelter behavioral evaluations, and contacted adopters of dogs from the Tompkins County SPCA in Ithaca, NY, USA. The shelter is no-kill, with open-admission and scheduled intake. Shelter programs to increase dog adoptions include: a small number of foster homes; a large number of volunteers who participate in dog walking, in-kennel training and companionship, overnight fostering of dogs, and day-trips with dogs; playgroups for suitable pairs of dogs; and adoption promotion via off-site events, social media, local print, and by a volunteer group independently advertising dogs that have been on the adoption floor for a long time. This research was conducted from August 2018 through January 2020 under protocol 2012-0150, which was approved by Cornell University’s Institutional Animal Care and Use Committee.

### 2.2. Dog Care and Housing

Upon intake, dogs were housed in the rescue building in chain link cages (indoor area, 2.2 m^2^ and outdoor run, 3.5 m^2^). All dogs were examined by veterinary staff at intake and received vaccinations, flea control, fecal exam, deworming, and a heartworm test. After the veterinary exam, each dog was scheduled for behavioral evaluation (Section 2.3). A day or two after behavioral evaluation, staff moved dogs to the adjacent pet adoption center where they were individually housed in one of 13 cubicles (from 5.2 m^2^ to 7.3 m^2^), which contained a raised bed, blanket, toys, and water bowl. Dogs were fed each day by staff between 08:00 and 09:00 h and again between 15:00 and 16:00 h. Staff and volunteers exercised dogs several times a day through leash walks and time in a large outdoor enclosure. Intact dogs were spayed or neutered before adoption.

### 2.3. Behavioral Evaluations

About 3 days after intake, each dog’s behavior was evaluated by two shelter staff (one serving as evaluator and one as scribe) using nine tests based on Sternberg’s Assess-a-Pet [12], with modifications described by Bollen and Horowitz [13]. Tests occurred in the following sequence: cage presentation; sociability; teeth exam; handling; arousal; food bowl; possession; stranger; and dog-to-dog. The Assess-a-Hand was used only during the food bowl test and possession test, and most dogs were tethered to the wall for these two tests. Dogs that displayed significant fear in response to tethering and extremely small dogs whose movements would be impeded by the heavy clip of the tether were held on a leash by the scribe. Responses scored for the food bowl and possession tests are shown in Table 1. As described in McGuire [3], we classified dogs as showing resource guarding if they exhibited at least one of the following behaviors during either the food bowl test, possession test, or both tests: stiffened, exhibited whale eye, snarled, froze, growled, lunged, snapped, or bit the Assess-a-Hand (food bowl test: responses 6 and 7; possession test: responses 4 and 5; Table 1). Source shelters transferring dogs to the Tompkins County SPCA only occasionally sent results from behavior assessments at their shelter; any results received were considered for that particular dog and the dog was tested by Tompkins’ staff as well.

### 2.4. Adopter Survey

Standard practice at the shelter (and continued during our study) is for the behavior or adoptions staff to counsel adopters of dogs either described by previous owners as resource guarding or assessed as resource guarding during a shelter behavioral evaluation. Staff follow a conversation-based adoption process during which adopters are fully informed about the guarding behavior reported by previous owners or observed by shelter staff and how it could impact the household. Staff also offer printed handouts and links to online videos with training tips (e.g., using positive reinforcement to teach “leave it” and “drop it”). It is also standard practice for a long-term volunteer to contact (via phone, email, or text) adopters of all dogs approximately 2 weeks after the dog entered its new home. The volunteer asks questions about how the dog is adjusting, whether there are any concerns, and if the dog has been examined by a veterinarian. Beginning in summer 2018, upon completion of these standard questions, the volunteer asked adopters if they would be willing to participate in our study on post-adoption behavior. We received the contact information for adopters who agreed to participate and then contacted them at least 4 weeks after the adoption (range, 4–12 weeks; pilot data indicated that response rates decreased after 12 weeks). We tried to reach each adopter via two of three different methods (phone, email, or text) before recording “no response”. We asked adopters to rank their dog’s behavioral responses from 0 to 2 (where 0 = no visible signs of aggression; 1 = growling or snarling; 2 = snapping or biting) in five different situations in which a family member: (1) took away toys, bones, or other objects valued by the dog (e.g., rawhides); (2) took away the dog’s food; (3) retrieved items or food taken by the dog; (4) approached the dog while it was eating; and (5) approached the dog at its favorite sleeping site (hereafter Q1–Q5; modified from the Canine Behavioral Assessment and Research Questionnaire, C-BARQ; [14] www.cbarq.org). We also asked three scripted questions (Q6–Q8; modified from Marder et al. [5]), beginning with whether the adopter considered their dog to be possessive in guarding food, toys, or space. If the adopter answered yes, then we asked whether they regarded their dog’s guarding behavior as a major concern and whether they felt it was difficult to prevent or manage possessive behaviors in their dog.

Of the 205 adopters who agreed to participate in our study when called by the shelter volunteer 2 weeks post adoption, 139 responded to our survey (58 by email; 53 by text and 28 by phone) for an overall response rate of 67.8%. Demographic data (age class and source) for the 139 dogs whose adopters responded are shown by sex in Table 2. Based on results of shelter behavioral evaluations, 20 of the 139 dogs (14.4%) were assessed as resource guarding and 119 (85.6%) as non-resource guarding. On average (mean ± *SD*), adopters responded 5.6 ± 1.4 weeks after adopting their dog. Time to respond did not differ between adopters of dogs assessed as resource guarding at the shelter (5.5 ± 1.6 weeks; range, 4–12 weeks) and adopters of dogs assessed as non-resource guarding (5.6 ± 1.1 weeks; range 4–11 weeks; *t* = 0.16, *d.f.* = 22.47, *p* = 0.87).

### 2.5. Canine Owner Surrender Profiles

We reviewed surrender profiles for 51 of the 57 dogs that were owner surrendered and whose adopters responded to our survey described in Section 2.4; owners of the remaining six dogs did not complete a surrender profile. We focused on three sections of the 4-page profile form that we considered potentially relevant to resource guarding behavior and scored each section as yes/no (Table S1). The first section asked, “Why are you surrendering your dog to the shelter?”, provided the options of behavioral problems, time commitment, family issues, health issues (owner), health issues (dog), and other, and instructed the owner to circle all that apply. We scored this as yes if the option, “behavioral problems”, was circled. The second section asked, “What does your dog do when you or someone else:” and listed nine scenarios from which we chose two: (1) “go near the food bowl?”; (2) “try to take away toys, rawhides, or anything else of value?”. We scored this as yes when at least one incident of growling, snarling, snapping, nipping, or biting was reported by surrendering owners. The scenarios not chosen in this section concerned responses to strangers in different settings and being hugged, reprimanded, and told to get off the sofa or bed. The third section began with, “Has your dog ever snarled at you or anyone else?” and provided lines where owners could check yes or no. In the four subsequent questions in this section, “snarled” was replaced with each of the following terms, respectively: “growled”, “snapped”, “nipped”, and “bitten (broken the skin)”. Owners were asked to explain the situation if they checked yes for any of the five behaviors. We scored this as yes when the owner reported the dog had displayed at least one of the five behaviors.

Of the 51 profiles available, 41 were mostly or fully completed (one question left unanswered or all questions answered). A few owner responses were either illegible or unclear in meaning and not included in the data set (e.g., an answer of “yes” or “no” to questions in the second section, such as “What does your dog do when you or someone else go near the food bowl?”). We made combined profiles for each of the six dogs that had more than one profile on file at the shelter during the time period of our study; these dogs had been returned to the shelter at least once. For combined profiles, we included all options circled on the different profiles as reasons for surrendering, and if a dog was reported as showing visible signs of aggression by one owner but not by another owner for questions in the second section (“What does your dog do when?”) and third section (“Has your dog ever?”), then we scored the dog as having shown visible signs of aggression.

### 2.6. Statistical Analyses

We used JMP Pro 13 for all statistical analyses.

#### 2.6.1. Relationships between Shelter Behavioral Evaluations and Adopter Reports

Relatively few adopters reported visible signs of aggression in their dogs at home, especially snapping or biting. Accordingly, we combined growling, snarling, snapping, and biting into a single category and scored adopter reports of their dog’s behavioral responses to the five situations described in our survey as visible signs of aggression reported or not reported. We present descriptive information on the specific behaviors shown by dogs. We excluded from analyses of adopter responses to survey questions, cases in which adopters did not place their dog in the particular situation described (e.g., adopters who did not take toys, bones, or other valued objects away from their dogs were excluded from the analysis of responses to Q1 and adopters who did not take food away from their dogs were excluded from the analysis of responses to Q2, etc.; exclusions are reflected in the denominators shown in Table 3, Section 3.1, and described in the footnote). We used Fisher’s exact test to examine whether resource guarding status of dogs based on shelter behavioral evaluations was associated with adopter reports of behavior in the home for the five situations surveyed (Q1–Q5). We also report positive predictive values and negative predictive values, as defined by Marder et al. [5], for each of the five questions. Positive predictive value is the likelihood that a dog that displayed food guarding during the shelter behavioral evaluation displayed food guarding in the adoptive home. Negative predictive value is the likelihood that a dog that did not display food guarding during the shelter behavioral evaluation did not display food guarding in the adoptive home. We include 95% confidence intervals for positive and negative predictive values.

#### 2.6.2. Relationships between Owner Surrender Profiles and Adopter Reports

We used logistic regression to determine whether different types of information from owner surrender profiles predicted adopter reports of visible signs of aggression in the home when toys, bones, or other valued objects were taken away by a family member (Q1 on the adopter survey). The model included the following predictor variables from surrender profiles: (1) behavioral problems indicated as a reason for surrender (yes/no); (2) resource guarding described (yes/no) in the section “What does your dog do when you or someone else:”; and (3) visible signs of aggression reported (yes/no) in the section, “Has your dog ever?”. For the second factor, we used information from the two scenarios (“What does your dog do when you or someone else goes near the food bowl” and “What does your dog do when you or someone else tries to take away toys, rawhides, or anything else of value”) to be consistent with our method of scoring dogs as resource guarding or non-resource guarding based on shelter behavioral evaluations (i.e., we classified dogs as resource guarding if they exhibited particular behaviors during either the food bowl test, possession test, or both tests). Sample sizes were too small for us to analyze whether information from the 51 surrender profiles predicted adopters responses to Q2 through Q5 of the survey because within the 51 surrendered dogs, the number reported by adopters to have shown visible signs of aggression was sometimes one or zero for these questions. As a result, models for Q2 through Q5 were unstable, so we only report results for Q1.

#### 2.6.3. Relationships between Shelter Behavioral Evaluations, Owner Surrender Profiles, and Adopter Reports

We compared shelter behavioral evaluations and owner surrender profiles in terms of prevalence of resource guarding and several measures of predictive ability with respect to behavior in the adoptive home (using responses to Q1 from the adopter survey). The following measures were defined in Patronek and Bradley [6] and we modified them for specific use with our data: sensitivity (proportion of adopted dogs that were correctly identified as resource guarding via the shelter evaluation or surrender profile); specificity (proportion of adopted dogs that were correctly identified as non-resource guarding via the shelter evaluation or surrender profile); false positive rate (proportion of adopted dogs identified by the shelter evaluation or surrender profile as resource guarding when they are not) and false negative rate (proportion of adopted dogs identified by the shelter evaluation or surrender profile as non-resource guarding when they are not). For shelter behavioral evaluations, we provide these five measures for all 139 dogs in the adopter survey. We also provide the five measures for only those dogs in the adopter survey that were owner surrendered to allow for a direct comparison between shelter behavioral evaluations and owner surrender profiles for the same group of dogs. For this comparison, we used two-sample proportion tests with a Yates’ continuity corrected to test whether measures of prevalence, sensitivity, specificity, false positive rate, and false negative rate were significantly different between the shelter behavioral evaluations and the owner surrender profiles. Finally, we had a complete owner surrender profile, shelter behavioral evaluation, and adopter report for a total of 44 dogs. We present in tabular form resource guarding status for these 44 dogs based on each of our three sources of information and provide Kappa statistics regarding levels of agreement between sources.

## 3. Results

### 3.1. Relationship between Shelter Behavioral Evaluations and Adopter Reports

Assessment of resource guarding at the shelter was significantly associated with adopter reports of dogs showing visible signs of aggression when toys, bones, or other valued objects were taken away in the home (*p* < 0.001; Q1, Table 3). The positive predictive value was 47.4% (PPV = 9/(9 + 10); 95% CI 27.3–68.3%) and the negative predictive value was 88.9% (NPV = 104/(104 + 13); 95% CI 81.9–93.4%). For dogs that showed resource guarding during the shelter assessment, eight adopters reported growling or snarling when valued objects were taken away; one adopter reported snapping or biting. For dogs that did not show guarding during the shelter assessment, ten adopters reported growling or snarling when valued objects were taken away; three reported snapping or biting.

Assessment of resource guarding at the shelter was significantly associated with adopter reports of dogs showing visible signs of aggression when food was taken away in the home (*p* < 0.001; Q2, Table 3). The positive predictive value was 33.3% (PPV = 6/(6 + 12); 95% CI 16.3–56.3%) and the negative predictive value was 97.4% (111/(3 + 111); 95% CI 92.5–99.1%). For dogs that showed resource guarding during the shelter assessment, five adopters reported growling or snarling when they took food away from their dog; one reported snapping or biting. For dogs that did not show guarding during the shelter assessment, three adopters reported growling or snarling when they took food away; none reported snapping or biting.

Assessment of resource guarding at the shelter was significantly associated with adopter reports of dogs showing visible signs of aggression when family members retrieved items or food taken by dogs (*p* < 0.02; Q3, Table 3). The positive predictive value was 23.5% (PPV = 4/(4 + 13); 95% CI 9.6–47.3%) and the negative predictive value was 95.5% (107/(5 + 107); 95% CI 90.0–98.1%). For dogs that showed resource guarding during the shelter assessment, four adopters reported growling or snarling when they retrieved items from their dog as did five adopters of dogs assessed as non-guarding. No adopters reported snapping or biting in this situation.

There was a tendency for assessment of resource guarding at the shelter to be associated with adopter reports of dogs showing visible signs of aggression when approached while eating in the home (*p* = 0.10; Q4, Table 3). The positive predictive value was 10.0% (2/(2 + 18); 95% CI 2.8–30.1%) and the negative predictive value was 98.3% (116/(2 + 116); 95% CI 94.0–99.5%). For dogs that showed resource guarding during the shelter assessment, two adopters reported growling or snarling when they approached their dog at the food bowl as did two adopters of dogs assessed as non-guarding. No adopters reported snapping or biting.

Assessment of resource guarding at the shelter was not associated with adopter reports of dogs showing visible signs of aggression when approached at a favorite sleeping site (*p* = 0.47; Q5, Table 3). The positive predictive value was 5.0% (PPV = 1/(1 + 19); 95% CI 0.9–23.6%) and the negative predictive value was 97.5% (NPV = 115/(3 + 115); 95% CI 92.8–99.1%). One adopter of a dog that displayed resource guarding during the shelter assessment reported growling or snarling when they approached the dog at a favorite sleeping site. For dogs assessed as non-guarding at the shelter, two adopters reported growling or snarling when they approached their dog at a favorite sleeping site and one reported snapping or biting.

Most adopters did not consider their dog to be possessive of food, toys, or space and classification as resource guarding at the shelter was not associated with adopters describing their dogs as possessive (*p* = 1.00; Q6, Table 4). Of those adopters who considered their dog possessive of food, toys, or space, most did not regard their dog’s guarding behavior as a major concern (Q7; Table 4) and most did not find it difficult to prevent or manage possessive behaviors in their dog (Q8, Table 4). Sample sizes were too small for formal statistical analysis of responses to Q7 and Q8.

### 3.2. Relationship between Owner Surrender Profiles and Adopter Reports

Of the 51 surrender profiles available, one was missing the page with the question, “Why are you surrendering your dog to the shelter?”; thus, the sample size was 50 profiles for this question. Table 5 shows the reasons owners provided for surrendering their dogs.

Forty-four of the 51 surrender profiles available had complete information for the section on resource guarding, with 70.5% (31/44) reporting no visible signs of aggression in their dogs and 29.5% (13/44) reporting at least one incident of growling, snarling, snapping, nipping, or biting when either the owner or someone else went near the food bowl or tried to take away toys, rawhides, or anything else of value (growling or snarling was reported in 11 dogs and snapping or nipping in two dogs).

Of the 51 surrender profiles available, 49 had complete information for the section on aggression, with 46.9% (23/49) reporting their dogs had never growled, snarled, snapped, nipped, or bitten the owner or anyone else and 53.1% (26/49) reporting their dogs had displayed at least one of the five behaviors listed (growling was the behavior most frequently reported by owners, 20 profiles, and biting was the behavior least frequently reported, one profile). Explanations supplied by owners for situations in which growling, snarling, snapping, nipping, or biting occurred typically fell into the following categories: resource guarding, aggression directed at either strangers or children, and handling sensitivities (e.g., growling during claw trimming).

Forty-four of the 51 profiles available had complete information for all three sections of interest (behavioral problems indicated as a reason for surrender, resource guarding described, and aggression described). Our logistic regression analysis revealed that description of resource guarding behavior in owner surrender profiles predicted visible signs of aggression when toys, bones, or other valued objects were taken away in the adoptive home (*X*^2^ = 5.57, *d.f.* = 1, *p* < 0.02; Table 6). The positive predictive value was 38.5% (5/(5 + 8); 95% CI 17.7–64.5%) and the negative predictive value was 93.6% (29/(2 + 29); 95% CI 79.3–98.2%). Citing behavioral problems as a reason for relinquishing a dog was not a significant predictor of visible signs of aggression when toys, bones, or other valued objects were taken away in the adoptive home (*X*^2^ = 0.44, *d.f.* = 1, *p* = 0.51; Table 6). The positive predictive value was 15.4% (2/(2 + 11); 95% CI 4.3–42.2%) and the negative predictive value was 83.9% (26/(5 + 26); 95% CI 67.4–92.9%). Similarly, description of visible signs of aggression in surrender profiles was not a significant predictor of visible signs of aggression when toys, bones, or other valued objects were taken away in the adoptive home (*X*^2^ = 0.06, *d.f.* = 1, *p* = 0.81; Table 6). The positive predictive value was 21.7% (5/(5 + 18); 95% CI 9.7–41.9%) and the negative predictive value was 90.5% (19/(2 + 19); 95% CI 71.1–97.3%).

### 3.3. Relationships between Shelter Behavioral Evaluations, Owner Surrender Profiles, and Adopter Reports

Table 7 shows prevalence and measures of predictive ability based on either shelter behavioral evaluations or owner surrender profiles in reference to behavior reported by adopters in survey Q1. For shelter behavioral evaluations, we provide values for both the total number of dogs in our study (*n* = 139) and for the subset of owner surrendered dogs for which we had complete data (*n* = 44). This allows a more direct comparison of the two sources of information—shelter behavioral evaluations and owner surrender profiles—for the same set of 44 dogs, although small sample size is problematic. Prevalence of resource guarding tended to be lower when based on shelter behavioral evaluations than when based on owner surrender profiles and measures of predictive ability were generally similar between these two sources of information (Table 7).

We had a complete owner surrender profile, shelter behavioral evaluation, and adopter report for 44 dogs (Appendix A). All three sources of information agreed with respect to resource guarding status (either yes or no) for 65.9% of dogs (29/44; dogs 1 through 29; Appendix A). Five of the 44 dogs, or 11.4%, were assessed as resource guarding by two of three sources (two dogs by surrendering owners and shelter; one dog by shelter and adopter; two dogs by surrendering owners and adopters; dogs 30 through 34; Appendix A). Ten of the 44 dogs, or 22.7%, were assessed as resource guarding by only one of the three sources (six dogs by surrendering owners; two dogs by shelter and two dogs by adopters; dogs 35 through 44; Appendix A). Finally, the level of agreement was slightly higher between shelter behavioral evaluations and adopter reports (*k* = 0.39; *p* = 0.01) than between either owner surrender profiles and adopter reports (*k* = 0.26; *p* = 0.06) or owner surrender profiles and shelter behavioral evaluations (*k* = 0.26; *p* = 0.06), although all three levels of agreement fell only in the fair range and Kappa can be less reliable when prevalence is low (Kappa of 1 = perfect agreement; 0 = agreement equivalent to chance; 0.21–0.40 = fair agreement; [15]).

## 4. Discussion

We found statistically significant associations between assessment as resource guarding during shelter behavioral evaluations and resource guarding reported in the adoptive home for three particular situations: taking away toys, bones or other valued objects; taking away food; and retrieving items or food taken by the dog. However, the positive predictive values for these three associations were low (47.4%, 33.3%, and 23.5%, respectively), meaning that from about one half to three quarters of dogs assessed as resource guarding in the shelter did not show guarding in these three situations in their adoptive homes. Thus, guarding behavior during the shelter assessment did not consistently indicate that guarding behavior would occur post adoption. Negative predictive values were high for these associations (88.9%, 97.4%, and 95.5%, respectively), indicating that almost all dogs assessed as non-resource guarding during shelter behavioral evaluations did not show guarding in these situations in their adoptive homes. Depending on the particular situation, from about 5–11% of dogs that did not show guarding during the shelter assessment were reported to show guarding post adoption. Results from shelter behavioral evaluations yielded a prevalence for resource guarding of 14.4% in our study population, which is similar to values reported for other shelter dog populations [2,4,5]. Conditions with low prevalence are associated with high negative predictive values and low positive predictive values [6], and our findings for resource guarding behavior fit this pattern.

Directly comparing our values with those from previous studies is somewhat challenging because adopters were asked slightly different questions and at different times post adoption. For example, we asked adopters separate questions about specific scenarios (e.g., taking away toys, bones or other valued objects; taking away food; and retrieving items or food taken by the dog) 4–12 weeks post adoption. Marder et al. [5] considered these scenarios (except the inclusion of toys) as one and categorized dogs as showing food aggression in the home when adopters reported visible signs of aggression over any of the following: a meal, delicious food items (such as bones and rawhides), or stolen food or table scraps; they surveyed adopters at least 3 months post adoption, with many adopters contacted more than 1 year after adoption. Nevertheless, our finding that 47.4% of dogs assessed as resource guarding in the shelter showed guarding in their adoptive homes when toys, bones, or other valued objects were taken away, is more similar to the 55% overall value reported by Marder et al. [5] and the 43% value reported by Van der Borg et al. [9] than the 10% value found by Mohan-Gibbons et al. [2]. One possible explanation for the lower percentage obtained by Mohan-Gibbons et al. [2] is that food aggressive dogs in their study were placed in a behavior modification program while in the shelter and also in their adoptive home, although compliance by adopters was low for at least some aspects of the program. Consistent with our prediction, assessment as resource guarding during either the food bowl test, possession test, or both tests was not associated with adopter reports of dogs showing visible signs of aggression when approached at a favorite sleeping site. This lack of an association likely reflects the sensitivity of resource guarding to context, setting, and type of resource [1,2,5]. This sensitivity was further demonstrated in our data by the differing proportions of dogs classified as resource guarding at the shelter that showed visible signs of aggression in the adoptive home across the five scenarios in our survey: 47.4% when toys, bones or other valued objects taken away; 33.3% when food taken away; 23.5% when taken items or food retrieved; 10.0% when approached while eating; and 5.0% when approached at a favorite sleeping site.

Regarding adopters’ perceptions of resource guarding behavior, we found that irrespective of whether their dog was classified as resource guarding or non-resource guarding at the shelter, most adopters (82–85%) did not consider their dog to be possessive of food, toys, or space. Of those adopters who considered their dog possessive of these items, most did not regard their dog’s guarding behavior as a major concern and did not find it difficult to prevent or manage possessive behaviors in their dog. Our findings on adopter perceptions of resource guarding agree with those described by Van der Borg et al. [9] and Marder et al. [5]. Mohan-Gibbons et al. [2] surveyed strength of bonds and found that most adopters of food guarding dogs described themselves as strongly bonded to their dog. These authors also reported that return rates at their study shelter for food guarding dogs were 5% as compared to 9% for the general dog population; it is worth noting, however, that food guarding dogs were screened to be highly adoptable based on scores from other tests in the shelter behavioral evaluation (i.e., having scores of one or two on other tests, indicating highly adoptable behavior, and scores of three, four, or five on the food bowl test, indicating stiff body language, growling, and attempting to bite). In a study of nearly 5 years of records from the Tompkins County SPCA, McGuire [3] differentiated resource guarding by level of severity, and found that guarding was a significant predictor of a dog being returned, with dogs that displayed severe guarding behaviors (lunging, snapping, biting) during the behavioral evaluation more likely to be returned (40.0%) than those showing mild to moderate guarding behaviors (e.g., growling, snarling, freezing; 18.2%) or no guarding behavior (17.5%). Given that dogs that show severe guarding behaviors typically make up 15–17% of resource guarding dogs at shelters [3,4], the majority of resource guarding dogs likely have return rates similar to those of non-resource guarding dogs. Additionally, many dogs that show severe guarding can be successfully placed, although more than one effort at re-homing may be needed [3]. Finally, we found that 11.1% of dogs assessed as non-resource guarding during the shelter behavioral evaluation showed guarding in their adoptive home; this value is somewhat lower than the 22% reported by Marder et al. [5]. Although larger sample sizes than we obtained are needed to fully address adopter attitudes regarding resource guarding (Table 4), it would be interesting to further examine whether attitudes differ between adopters who knowingly take home dogs assessed as resource guarding at shelters and find continued guarding in the home and adopters who take home dogs assessed as non-resource guarding at shelters and see guarding behavior in the home.

For the subset of dogs in our adopter survey that were relinquished by owners, we examined information provided by their owners on three parts of the shelter’s surrender profile form: reasons for surrendering the dog; questions about resource guarding; and questions about visible signs of aggression toward either the owner or another person. Reasons for surrender to the Tompkins County SPCA included both human-related reasons (family issues; housing issues, which fell under “other”; time commitment; and health) and dog-related reasons (behavioral problems; health; space needs; and inability to get along with resident pets), as has been found in previous studies of dogs surrendered to shelters [16,17,18,19]. With respect to questions on resource guarding, 29.5% of owners reported at least one incident of growling, snarling, snapping, nipping, or biting when the owner or someone else either went near the food bowl or tried to take away toys, rawhides, or anything else of value. For questions about whether dogs had ever growled, snarled, snapped, nipped, or bitten the owner or anyone else, 53.1% of owners reported their dogs had displayed at least one of the five behaviors listed, with growling most frequently reported, and biting least frequently. Of the responses provided by owners to questions in these three sections of the surrender profile, only responses to questions about resource guarding behavior significantly predicted guarding in the adoptive home. However, the positive predictive value was 38.5% and the negative predictive value was 93.6%, suggesting that owner responses on the surrender profile indicating absence of guarding behavior in their dog may be more informative than owner responses indicating presence of guarding behavior. Stephen and Ledger [20] found information provided by surrendering owners to be of limited usefulness in predicting behavior in adoptive homes: eight of the 20 behaviors described by relinquishing owners were significantly correlated with incidence of these behaviors in the adoptive home at 2 weeks post adoption and six of the 20 were significantly correlated at 6 weeks post adoption (most correlation coefficients for these 14 behaviors were between 0.4 and 0.7, typically considered the moderate rather than high range). Unfortunately, resource guarding was not one of the 20 behaviors monitored. Possible explanations for dogs behaving differently in successive households include differences in characteristics of owners, adopters, and households and the intervening experience of living at an animal shelter [20].

Our measures of predictive abilities of the resource guarding component of shelter behavioral evaluations at the Tompkins County SPCA, in reference to subsequent behavior in adoptive homes (Table 7, column 2), are similar to those obtained by Marder et al. [5] and summarized in Patronek et al. [7] Figure 1. For example, the following values were obtained by us and by Marder et al. [5], respectively: sensitivity (40.9%, 39.3%); specificity (91.2%, 87.0%); false positive rate (52.6%, 45.0%); and false negative rate (11.1%, 22.1%). These similarities are interesting given differences in study shelters, behavioral evaluations, survey methods, and potential differences in dog populations, although additional values from other studies of resource guarding are needed to know if these measures are typical. We also examined owner surrender profiles, which allowed us to directly compare measures of prevalence and predictability between shelter behavior evaluations and surrender profiles for the same 44 dogs (a subset of those in the adopter survey; Table 7, columns 3 and 4). Unfortunately, these comparisons were compromised by small sample sizes and additional data are needed to draw firm conclusions. Nevertheless, the prevalence data for this subset of dogs (shelter behavioral evaluations, 18.2%, versus owner surrender profiles, 29.5%) suggested that owners were not under reporting resource guarding behavior by their dogs at time of surrender as has been suggested for owner-directed aggression and fear of strangers [11]. Finally, our data allowed us to compare levels of agreement between the three sources of information—owner surrender profiles, shelter behavioral evaluations, and adopter reports—for these 44 dogs. We found that level of agreement was slightly higher between shelter behavioral evaluations and adopter reports than between either owner surrender profiles and adopter reports or owner surrender profiles and shelter behavioral evaluations, although all three levels of agreement were in the fair range.

Limitations of our study include sample size issues linked, in part, to the fairly low prevalence of resource guarding in shelter dogs. Relatively few dogs in shelters display resource guarding during behavioral evaluations (average prevalence of 14% across 77 shelters in the United States, range 7–30%; [2]). Prevalence at our study shelter was 14.4% and this made it challenging for us to obtain sufficient numbers of resource guarding dogs. Over our 1.5 year study period, we were able to gather data from adopters of 20 dogs assessed as resource guarding in the shelter; adopters of another 11 dogs assessed as resource guarding agreed to participate in our survey but did not respond. Another limitation is that we considered as one group dogs that were tethered to the wall and dogs held on a leash by a staff member during the food bowl and possession tests at the shelter. Most dogs were tethered to the wall and the decision to hold a dog on a leash was made either for humane reasons (dogs showed significant fear of tethering) or test accuracy (the heavy clip of the tether inhibited movements of very small dogs). Nevertheless, dogs are sensitive to environmental context, and tethering/not tethering could have influenced their behavior during tests. We contacted adopters 1–3 months after adoption because pilot data indicated lower response rates after 3 months. While our time frame might seem too soon to assess a dog’s behavior in a new home, our results were very similar to those of Marder et al. [5], who surveyed adopters at least 3 months postadoption and typically one or more years after adoption. Finally, over a nearly 5 year period (2014–2019) that partially overlapped with the current study, about 9% of dogs assessed as resource guarding at the Tompkins County SPCA were not made available for adoption; some were euthanized for behavioral reasons while others were transferred to rescue groups or returned to the owner [3]. Thus, it is possible that some of the dogs most likely to show resource guarding in an adoptive home were removed from the shelter population before reaching the adoption floor.

## 5. Conclusions

Although we found statistically significant associations between resource guarding during shelter behavioral evaluations and resource guarding reported in adoptive homes for three of five situations surveyed, positive predictive values were low, with at least half of dogs assessed as resource guarding in the shelter not showing guarding in adoptive homes. Our results, together with those from other research studies [2,3,4,5,9,10] and analyses put forth in critiques of shelter behavioral assessments [6,7] call into question the practice in some U.S. shelters of considering all dogs assessed as food aggressive on behavioral evaluations to be unadoptable and candidates for euthanasia. We also found that owner responses on surrender profiles to specific questions concerning resource guarding significantly predicted guarding in adoptive homes. Again, however, the positive predictive value was low, with more than half of dogs described as resource guarding by surrendering owners not showing guarding in adoptive homes. The negative predictive value was quite high (almost 94% of dogs described as non-guarding by surrendering owners did not show guarding post adoption), demonstrating the continued importance for shelters to collect information from surrendering owners, even if the information concerns behaviors not shown by the dog. For presence or absence of resource guarding behavior in the subset of owner-surrendered dogs, our measures of agreement from two-way comparisons of owner surrender profiles, shelter behavioral evaluations, and adopter reports fell in the fair range, perhaps reflecting the complexity of resource guarding behavior and importance of the setting in which it is evaluated. A source of information not studied here that might aid in predicting behavior in adoptive homes, is reporting from experienced staff or volunteers who temporarily foster a shelter dog in their home. We are unaware of any studies on the predictive abilities of reports from experienced fosterers in the context of behavior post adoption and encourage such research.

## Figures and Tables

**Table 1:** Possible responses during food bowl and possession tests on the canine behavioral evaluation at the shelter ^1^.

Response	Food Bowl Test	Possession Test
1	Stopped eating and backed away from dish	Readily dropped item
2	Continued eating without signs of uneasiness	Allowed Assess-a-Hand to take item
3	Moved muzzle deeper into dish and ate faster	Resisted letting go of item but did not show outward aggression
4	Stiffened slightly	Stiffened, exhibited whale eye, snarled
5	Moved muzzle toward Assess-a-Hand	Froze, growled, lunged, snapped, bit Assess-a-Hand
6	Stiffened, exhibited whale eye, snarled	-----
7	Froze, growled, lunged, snapped, bit Assess-a-Hand	-----

^1^ Kibble and canned food were provided during the food bowl test and a food-related item, such as a raw hide chew or pig’s ear, was provided during the possession test.

**Table 2:** Demographic data for dogs (*n* = 139) whose adopters responded to our survey.

Demographic Information	Males	Females
Age class ^1^		
Juvenile	13	11
Adult	54	39
Senior	12	10
Source		
Surrendered by owner	32	25
Transferred from another shelter	24	22
Picked up as a stray	11	5
Returned by adopter	7	8
Seized by animal control officer	5	0

^1^ Juveniles, from 4 months to <1 year; adults, from 1 year to <8 years; and seniors, ≥8 years. We did not track behavior in the adoptive home for puppies because shelter behavioral evaluations for this age group differ from those for dogs at least 4 months of age.

**Table 3:** Responses of adopters to survey questions about how their dog responds to five different situations in the home (Q1–Q5). Dogs are classified as resource guarding (*n* = 20) or non-resource guarding (*n* = 119) based on behavioral evaluations at the shelter before adoption.

Adopter Survey Questions and Resource Guarding Status of Adopted Dogs	No Visible Signs of Aggression Reported ^1^	Visible Signs of Aggression Reported ^1^
Q1: Toys, bones or other valued objects taken away?		
Resource guarding	52.6% (10/19)	47.4% (9/19)
Non-resource guarding	88.9% (104/117)	11.1% (13/117)
Q2: Food taken away?		
Resource guarding	66.7% (12/18)	33.3% (6/18)
Non-resource guarding	97.4% (111/114)	2.6% (3/114)
Q3: Taken items or food retrieved?		
Resource guarding	76.5% (13/17)	23.5% (4/17)
Non-resource guarding	95.5% (107/112)	4.5% (5/112)
Q4: Approached while eating?		
Resource guarding	90.0% (18/20)	10.0% (2/20)
Non-resource guarding	98.3% (116/118)	1.7% (2/118)
Q5: Approached at a favorite sleeping site?		
Resource guarding	95.0% (19/20)	5.0% (1/20)
Non-resource guarding	97.5% (115/118)	2.5% (3/118)

^1^ Percentages represent number of dogs reported by adopters to either not show or show visible signs of aggression divided by number of dogs put in each situation (e.g., For Q1, one adopter of a dog classified as resource guarding at the shelter and two adopters of dogs classified as non-resource guarding at the shelter did not take toys, bones, or other valued objects away from their dogs and for Q2, two adopters of dogs classified as resource guarding at the shelter and five adopters of dogs classified as non-resource guarding at the shelter did not take food away from their dogs). Visible signs of aggression included growling, snarling, snapping, or biting.

**Table 4:** Responses (no/yes) of adopters to the three scripted survey questions (Q6–Q8). Dogs are classified as resource guarding (*n* = 20) or non-resource guarding (*n* = 119) based on behavioral evaluations at the shelter before adoption.

Adopter Survey Questions and Resource Guarding Status of Adopted Dogs	No	Yes
Q6: Consider your dog to be possessive in guarding food, toys, or space?		
Resource guarding	85.0% (17/20)	15.0% (3/20)
Non-resource guarding ^1^	82.2% (97/118)	17.8% (21/118)
Q7: If yes to Q6, regard your dog’s guarding behavior as a major concern?		
Resource guarding	100.0% (3/3)	0.0% (0/3)
Non-resource guarding	71.4% (15/21)	28.6% (6/21)
Q8: If yes to Q6, difficult to prevent or manage possessive behaviors in your dog?		
Resource guarding	100.0% (3/3)	0.0% (0/3)
Non-resource guarding	76.2% (16/21)	23.8% (5/21)

^1^ One adopter of a dog classified as non-resource guarding at the shelter left this question blank; thus, sample size is 118 for non-resource guarding dogs rather than 119.

**Table 5:** Reasons provided by owners for surrendering their dog to the shelter, ranked from highest to lowest. Owners were asked to circle all that apply, so percentages do not add to 100%.

Reason for Surrender	Percentage of Surrender Profiles (*n* = 50)
Family issues	38% (19/50)
Other ^1^	28% (14/50)
Behavioral problems	26% (13/50)
Owner’s health	22% (11/50)
Time commitment	18% (9/50)
Dog’s health	8% (4/50)

^1^ For profiles in which the option “other” was circled, the reasons provided were owner housing issues (*n* = 7; moving, military deployment, eviction, and homelessness), dogs either not getting along with resident pets (*n* = 2) or needing more space (*n* = 2), inability to pay veterinary expenses (*n* = 1), and owner deceased (*n* = 1). One owner provided no explanation.

**Table 6:** Relationship between owner-supplied information at the time of canine surrender to the shelter and adopter reports of resource guarding behavior in the new home when toys, bones, or other valued objects were taken away (Q1 on the adopter survey).

Owner-Supplied Information on Canine Surrender Profile ^1^	Adopter Report of No Visible Signs of Aggression When Valued Objects Taken Away	Adopter Report of Visible Signs of Aggression When Valued Objects Taken Away
Behavioral problems circled		
Yes	84.6% (11/13)	15.4% (2/13)
No	83.9% (26/31)	16.1% (5/31)
Resource guarding described		
Yes	61.5% (8/13)	38.5% (5/13)
No	93.6% (29/31)	6.4% (2/31)
Signs of Aggression described		
Yes	78.3% (18/23)	21.7% (5/23)
No	90.5% (19/21)	9.5% (2/21)

^1^ A response of yes to owner-supplied information indicates the following: (1) In response to the question, “Why are you surrendering your dog to the shelter?”, the owner circled the option behavioral problems (other options also could be circled); (2) In response to the question, “What does your dog do when you or someone else go near the food bowl or try to take away toys, rawhides, or anything else of value?”, the owner described at least one incident in which the dog growled, snarled, snapped, nipped, or bit; and (3) In response to the questions, “Has your dog ever snarled (or growled, snapped, nipped, bitten) at you or anyone else?”, the owner responded yes to at least one of the five behaviors.

**Table 7:** Measures of prevalence and predictive abilities for shelter behavioral evaluations and owner surrender profiles with respect to behavior shown by dogs in adoptive homes when toys, bones, or other valued objects were taken away (Q1 on adopter survey). Measures for shelter behavioral evaluations are shown for all dogs included in the adopter survey (*n* = 139; column 2) and for only those dogs that were owner surrendered and for which we had complete data (*n* = 44; column 3). *p* value is from comparison of values in columns 3 and 4.

Measures ^1^	Shelter Behavioral Evaluation (All Dogs)	Shelter Behavioral Evaluation (Only Owner Surrendered Dogs)	Owner Surrender Profile	*p*
Prevalence	14.4% (20/139)	18.2% (8/44)	29.5%(13/44)	0.32
Sensitivity	40.9% (9/22)	50.0% (4/8)	71.4% (5/7)	0.75
Specificity	91.2% (104/114)	88.9% (32/36)	78.4% (29/37)	0.37
False positive rate	52.6% (10/19)	50.0% (4/8)	61.5% (8/13)	0.95
False negative rate	11.1% (13/117)	11.1% (4/36)	6.5% (2/31)	0.81

^1^ Measures based on Patronek and Bradley [6] and defined as follows: prevalence (number of dogs that either displayed resource guarding during the shelter behavioral evaluation or were described by owners as resource guarding at time of surrender to the shelter/total number of dogs either evaluated or surrendered); sensitivity (proportion of adopted dogs that were correctly identified as resource guarding via the shelter evaluation or surrender profile); specificity (proportion of adopted dogs that were correctly identified as non-resource guarding via the shelter evaluation or surrender profile); false positive rate (proportion of adopted dogs identified by the shelter evaluation or surrender profile as resource guarding when they are not) and false negative rate (proportion of adopted dogs identified by the shelter evaluation or surrender profile as non-resource guarding when they are not).

## Supplementary Materials

The following is available online at https://www.mdpi.com/2076-2615/10/9/1702/s1, Table S1: Questions selected for analysis from three sections of the Tompkins County SPCA’s Owner Surrender Profile Form. Our method of scoring owner responses is shown in parentheses.

## Appendix A

**Table A1:** A comparison of resource guarding status based on owner surrender profiles, shelter behavioral evaluations, and adopter reports for 44 dogs. Shaded cells with a Y (for Yes) indicate resource guarding was reported by surrendering owner, shelter evaluator, or adopter. Unshaded cells with an N (for No) indicate no resource guarding was reported.

Dog	Owner Surrender Profile	Shelter Behavioral Evaluation	Adopter Report
1	Y	Y	Y
2	Y	Y	Y
3	Y	Y	Y
4	N	N	N
5	N	N	N
6	N	N	N
7	N	N	N
8	N	N	N
9	N	N	N
10	N	N	N
11	N	N	N
12	N	N	N
13	N	N	N
14	N	N	N
15	N	N	N
16	N	N	N
17	N	N	N
18	N	N	N
19	N	N	N
20	N	N	N
21	N	N	N
22	N	N	N
23	N	N	N
24	N	N	N
25	N	N	N
26	N	N	N
27	N	N	N
28	N	N	N
29	N	N	N
30	Y	Y	N
31	Y	Y	N
32	N	Y	Y
33	Y	N	Y
34	Y	N	Y
35	Y	N	N
36	Y	N	N
37	Y	N	N
38	Y	N	N
39	Y	N	N
40	Y	N	N
41	N	Y	N
42	N	Y	N
43	N	N	Y
44	N	N	Y
