# Supplementary material for: Abilities of Canine Shelter Behavioral Evaluations and Owner Surrender Profiles to Predict Resource Guarding in Adoptive Homes
Source: Animals (Basel). 2020 Sep 20;10(9):1702. doi: 10.3390/ani10091702 (PMC7552731; doi:10.3390/ani10091702)
Supplement: Supplementary file 1 [file animals-10-01702-s001.pdf]

Table S1. Questions selected for analysis from three sections of the Tompkins County SPCA's Owner Surrender Profile Form. Our method of scoring owner responses is shown in parentheses.

Section 1: Reason for surrendering

Why are you surrendering your dog to the shelter (Circle all that apply)?
Options provided: behavioral problems, time commitment, family issues, health issues (owner), health issues (dog), and other.
(We scored this as yes when the option behavioral problems was circled by surrendering owners.)

Section 2: Resource guarding

What does your dog do when you or someone else:
go near the food bowl?____________________
try to take away toys, rawhides, or anything else of value?____________________
(We scored this as yes when at least one incident of growling, snarling, snapping, nipping, or biting was reported by surrendering owners.)

Section 3: Visible signs of aggression

Has your dog ever snarled at you or anyone else? Yes______No_____If yes, please explain the situation.
____

Has your dog ever growled at you or anyone else? Yes_____No_____If yes, please explain the situation.
____

Has your dog ever snapped at you or anyone else? Yes_____No_____If yes, please explain the situation.
____

Has your dog ever nipped at you or anyone else? Yes______No_____If yes, please explain the situation.
____

Has your dog ever bitten (broken skin) you or anyone else? Yes_____No_____If yes, please explain the situation.
____

(We scored this as yes when the owner reported the dog had displayed at least one of the five behaviors.)
